# Genetic Counsellors play a key role in supporting ethically responsible expanded universal carrier screening

**DOI:** 10.1038/s41431-022-01218-3

**Published:** 2022-11-07

**Authors:** Lisa Dive, Lucinda Freeman, Alison McEwen

**Affiliations:** Graduate School of Health, University of Technology Sydney, Broadway, NSW Australia

**Keywords:** Ethics, Psychology

Commentary on: “Societal implications of expanded universal carrier screening: a scoping review” van den Heuvel et al. [1]

Since the first human genome was sequenced in 2003 [2], the cost of sequencing a human genome has gone from approximately US$100,000,000.00 in 2003 to US$1000 in 2021 [3], and the time required has drastically reduced. Coupled with these spectacular technological advances, there is increasing—although still highly incomplete—knowledge about the (causal) role that variants in different genes play in the development of many different health conditions. In a process known as ‘carrier screening’, it is now possible for a pregnant (or not yet pregnant) couple to screen their own genes to identify the chance that, together, they could have children with a genetic condition. If they are found to have an increased chance, then the couple may choose to take various courses of action to avoid having a child with that condition.

Carrier screening for reproductive purposes is often called reproductive genetic carrier screening (RGCS) or expanded universal carrier screening (EUCS), because it screens for a large (expanded) range of conditions, and is sometimes universally available. There have been concerns that population level RGCS has similarities to abhorrent eugenics programs in the past. To ensure the ethical acceptability of RGCS it is important that participation is optional, and that the primary goal is to support couples’ reproductive autonomy [4]. There is great optimism that a considered and ethically robust offer of RGCS has the capacity to reduce significant suffering associated with severe and devastating genetic conditions [5].

The test results of RGCS can be unfamiliar, complex or ambiguous, generating a range of ethical considerations. Many of the conditions included on screening panels are rare, meaning that the couple, and likely their healthcare provider, may be unfamiliar with condition. The couple might not know if they think that the genetic condition identified warrants costly and burdensome reproductive interventions with low chances of success. Alternatively, the couple might want to avoid the condition but cannot afford IVF and preimplantation genetic testing (PGT); the couple might be pregnant at the time of screening and go on to learn that their fetus is affected by a serious genetic condition, but termination of pregnancy is not available in the jurisdiction where they live. Other couples might not think that knowledge of their carrier status would affect their reproductive choices. If RGCS becomes routinised, will they be able to decline an offer of screening without being condemned as bad (future) parents? If they go on to have a child with a condition for which carrier screening was available, will they be able to access appropriate healthcare for the child?

Access to RGCS, both through government funded population screening programs and through user-pays commercial offerings, brings broad societal challenges. There is the risk that health inequities will deepen, and people who are already vulnerable will be further disadvantaged. If RGCS becomes widely available, the inherent complexities and ambiguities of this kind of screening will require a reflective, cross-disciplinary approach. Access to adequate information and decision-making support and time to reflect prior to accepting an offer of RGCS is crucial. The type of support needed is likely to vary across demographics that include geographic location, ethnicity, health literacy and language spoken. Disability advocacy groups have valid concerns that healthcare and societal attitudes towards people living with disabilities will be impacted if RGCS becomes widespread and routinised.

If RGCS is offered without attention to its social context, it has the potential to cause significant harms. Therefore, the scoping review of the societal implications of RGCS or EUCS is extremely timely (van den Heuvel [1]). Several jurisdictions are trialling publicly funded or subsidised offers of RGCS [6, 7], and many private companies are offering it on a user-pays basis. As RGCS becomes more widespread, its potential to influence societal norms—either in positive or negative ways—becomes more real.

One of the most significant consequences of a universal offer of expanded carrier screening that van den Heuvel et al. [1] identify is the potential for routinisation. Routinisation occurs when an intervention is considered to be part of the routine standard of care and tends to carry a normative weight; in other words, taking up an offer of screening could become perceived as the right thing to do, or what a good, responsible prospective parent ought to do [8]. A consequence of routinisation is that societal norms come to undermine reproductive autonomy, because prospective parents do not have a genuine option to decline screening. If an intervention is perceived as routine, then a couple who does not consider that it has value for them might still feel pressure to undertake screening.

Insights from the clinical practice of genetic counsellors reveal strategies that can minimise potential harms arising from RGCS, and ensure that the way screening is offered supports its intended aims—namely, to provide information that is valuable for couples in the context of their reproductive decision making. Genetic counsellors have a role to play both in supporting couples who are making reproductive decisions, and through their roles as educators of healthcare professionals and communities. Engaging in policy decisions offers an additional opportunity for genetic counsellors to minimise the potential harms as RGCS becomes more widely available.

As van den Heuvel et al. [1] emphasise, the way EUCS is implemented will have the greatest impact on whether certain (positive or negative) potential societal impacts actually occur. As such, the perspective of clinical genetic counselling has much to offer to support a socially responsible and ethically acceptable offer of carrier screening that upholds a genuine commitment to reproductive autonomy as its primary goal. Integrating perspectives and approaches from genetic counselling can support prospective parents to engage in a reflective questioning of whether EUCS is right for them. For example, in the reproductive genetic carrier screening pilot project in Australia (Mackenzie’s Mission), an interactive decision aid—informed by a range of experts in fields including clinical genetics, genetic counselling and bioethics—was developed to support potential participants to reflect on whether carrier screening aligned with their preferences and values [9].

An “increased chance” result from EUCS has a cascade of impacts on the carrier couple, their reproductive choices, and also potentially their wider families. The unique skills of genetic counsellors are crucial to support couples who receive a result that shows they are at increased chance of having children with a genetic condition. Following such a result couples should ideally have access to genetic counselling provided by genetic counsellors to understand the result and what it means for them and their family, and to clarify if and how the result might influence their reproductive choices. Given the complexity of many genetic test results it is essential that screening not be offered without access to adequate support after a carrier finding [10].

In addition to the shorter-term impact on reproductive decision making, a carrier screening result has the potential to trigger cascade genetic testing among other family members. Ensuring an adequate workforce is available to manage the flow-on impact of increased identification of carriers of genetic conditions is an important consideration in the planning of widespread access to RGCS.

As technological advances and reduced costs make RGCS a feasible option in some jurisdictions, the review by van den Heuvel et al. [1] reveals the importance of a considered approach to how such screening is offered. Widespread availability of RGCS raises many ethical considerations, and the way it is offered will have a significant impact on our society. Routinisation is a key concern, as the ethical implementation of RGCS requires that there be a genuine option to decline screening or further intervention following a screening result. Genetic counsellors and other healthcare providers, along with consumer and community groups, have a central role in leading the discussion that is needed prior to implementing RGCS. Drawing on relevant clinical perspectives can inform the development and implementation of ethically robust offerings of RGCS that go some way towards responding to the societal ethical concerns identified in this review.
